# Digital pathology: Revolutionizing oral and maxillofacial diagnostics

**DOI:** 10.6026/9732063002001834

**Published:** 2024-12-31

**Authors:** Mrunali Ghanasham Gharat, Sneha Masne Deshpande, Swati Dhone, Vibhuti Shreesh Mhatre, Bhavani Nagendra Sangala, Jyotsna Sethumadhavan, Amit Patil, Prachi Gholap

**Affiliations:** 1 Department of Oral Pathology and Microbiology Bharati Vidyapeeth (Deemed to be University) Dental College and Hospital, Belapur, Navi Mumbai - 400614, Maharashtra, India; 2 Department of Pediatric and Preventive Dentistry, Bharati Vidyapeeth (Deemed to be University) Dental College and Hospital, Belapur, Navi Mumbai - 400614, Maharashtra, India; 3 Department of Oral and Maxillofacial Pathologist, Oral Pathology and Microbiology, YMT Dental College and Hospital, Navi Mumbai, Maharashtra, India; 4 Department of Prosthodontics, Crown and Bridge, Bharati Vidyapeeth (Deemed to be University) Dental College and Hospital, Belapur, Navi Mumbai, Maharashtra - 400614, India; 5 Department of Conservative Dentistry & Endodonotics, Bharati Vidyapeeth Deemed to be University Dental College and Hospital, Navi Mumbai, Maharashtra - 400614, India

**Keywords:** Computational pathology, digital slide scanning, machine learning in pathology, maxillofacial diagnostic precision

## Abstract

Digital pathology (DP) has revolutionized oral and maxillofacial pathology (OMP) by enhancing diagnostic accuracy and efficiency, leading to improved patient outcomes. Therefore, it is of interest to report on the potential of Whole Slide Imaging (WSI), Artificial Intelligence (AI) and telepathology in OMP, highlighting their role in facilitating remote consultations and automated image analysis. AI and Machine Learning (ML) have further advanced cancer diagnosis by improving pattern recognition and predictive accuracy. While Digital pathology offers numerous benefits, challenges such as data management and ethical considerations remain. Future research should explore ways to further integrate Digital pathology into OMP practice.

## Background:

The term Digital pathology refers to the transmission of a pathologist's diagnosis over a data link that enables the transfer of images and real-time video recordings, along with specific digital images of macroscopic and microscopic preparations, clinical information and case specific data [1]. The field of digital OMP encompasses the use of advanced technology to diagnose and analyze diseases affecting the mouth, jaw and surrounding structures also; many benefits of switching to Digital pathology over traditional light microscopy have been demonstrated by a substantial body of research, including increased efficiency, quality and safety in pathology labs. The development of Digital pathology has nearly eliminated the need for "conventional" telepathology [2]. Services for diagnosis and consultation made up the majority of the goal. Digital diagnostic pathology practice may benefit from the use of 3D imaging technology in the future, as it can generate simulations at the microscopic level [3]. The objective of this review article is to give readers a thorough understanding of digital technologies' present and potential applications in OMP which includes examining the impact of Digital pathology on diagnostic accuracy, efficiency and patient outcomes, as well as exploring the challenges and limitations associated with its implementation. Therefore, it is of interest to describe how digital pathology has revolutionized oral and maxillofacial pathology by enhancing diagnostic accuracy, efficiency and patient outcomes and to explore the transformative role of innovations such as Whole Slide Imaging (WSI), Artificial Intelligence (AI) and telepathology in shaping the future of the field.

## Traditional methods in oral and maxillofacial pathology (OMP):

## Digital pathology (DP):

This workflow comprises digital image analysis, digital dictation, digital dictation and speech recognition tools and laboratory information management systems in addition to digital reporting [2]. It is common to mention consultation pathology as the perfect use case for Digital pathology. The most popular use of Digital pathology to date may be in medical education and it's a rather simple use. For training reasons, dedicated digital microscopes for presentations can stream live video and display the slide review process, providing insight into the histopathological evaluation approach. Digital photos can be utilized for tests, annotated in presentations and made available for independent and remote study [4]. Digital pathology's inherent significance stems from its capacity to surmount the constraints linked to traditional methods. Pathology has advanced significantly with this paradigm change. It transforms the tiny world into a digital format, embracing the digital era and moving beyond actual glass slides. Whole Slide Imaging is the foundational idea of Digital pathology. Glass slides are converted into high-resolution digital images using Whole Slide Imaging, which makes it possible to store and share data with ease and do sophisticated analysis [5].

## Applications:

Conferences, diagnosis, education, patient understanding, when teaching oral pathology through virtual microscopy, dental student's exhibit great compliance, making this an effective educational tool [6]. Technological innovations in digital oral and maxillofacial pathology (OMP). The studies highlighting key applications of DOMP are summarised in Table 1 Studies highlighting key applications of DOMP.

## Whole Slide Imaging (WSI):

Since its initial introduction around twenty years ago, Whole Slide Imaging has been verified for numerous pathology applications. The recent authorization from the US FDA for a Whole Slide Imaging system to be used in primary surgical pathology diagnosis has created opportunities for broader adoption and utilization of this technology in everyday practice [7]. Having digital copies reduces the chances of damaging the original samples and facilitates the distribution of copies among other pathologists [8]. The field of pathology, like most medical specialties, is currently under pressure to enhance quality, patient safety and diagnostic precision due to the increasing focus on sub specialization. These factors, combined with financial pressures to consolidate and centralize diagnostic services, are propelling the creation of systems that can enhance access to expert opinions and highly specialized pathology services. Digital pathology networks utilizing Whole Slide Imaging systems offer a potential solution to these challenges and will unquestionably have a crucial role to play in the future [9]. The steps that are included in Whole Slide Imaging are summarised in Figure 1(see PDF) Steps in Whole Slide Imaging. Figure 2(see PDF) shows AI's contributions to diagnostic imaging, with major impacts in predictive analytics (33%), complex procedure assistance (30%), enhanced image analysis (20%) and efficiency (23%) and smaller roles in reducing errors (17%), integration (3%), personalized medicine (3%) and cost-effectiveness (3%). AI and ML have become integral components in Digital pathology, revolutionizing the way pathologists analyze and interpret histo-pathologic images. The studies in which AI and pathology are integrated are listed in Table 2 Studies highlighting integration of AI and pathology. The use of AI and deep learning algorithms has shown great promise in enhancing diagnostic accuracy, improving biomarker assessment and enabling personalized therapy decisions in various areas of pathology, including breast cancer, prostate cancer and hematolymphoid disorders.

## Workflow:

## The workflow in Digital pathology typically involves several key steps:

This workflow enhances the effectiveness and precision of pathology diagnostics, ultimately improving patient care [28]. Figure 3(see PDF) diagram shows the Digital pathology workflow, from slide scanning to Whole Slide Imaging creation, analysis, report compilation and system integration into clinical workflows.

## Telepathology:

Telepathology in OMP is an emerging field that utilizes technology to enhance the teaching, the identification, assessment and management of diseases and abnormalities in the mouth and jaw area [29].

[1] Shared their experience in setting up a virtual microscope and telepathology system for the Oral and Maxillofacial Pathology (OMP) laboratory course at a dental school.

[2] The system allowed students to report and discuss histological findings of virtual teaching slides with tutors in a web-based computer classroom.

This approach not only facilitated learning but also provided a platform for evaluation and feedback. The second-opinion diagnosis patterns in OMP are being examined and highlighting the importance of seeking additional perspectives in complex cases [30]. This emphasizes the role of telepathology in facilitating collaboration and knowledge sharing among pathologists, ultimately leading to more, diagnoses and treatment plans that are precise and effective [31]. Explored the role of diagnostic ultrasound is new diagnostic aid in oral and maxillofacial surgery. While still in its early stages, this technique shows promise in diagnosing various tissue pathologies in the field of oral surgery. Integrating diagnostic ultrasound with telepathology systems could potentially enhance the accuracy and efficiency of diagnosing oral and maxillofacial pathologies. In a retrospective study by [32], pediatric oral biopsies were evaluated to understand the prevalence and characteristics of oral and maxillofacial lesions in the pediatrics. The majority of lesions detected were benign, underscoring the importance of accurate diagnosis and management, which can be facilitated through telepathology systems. 3D models have significant influence in oral and maxillofacial surgery and ability to enhance surgical results and patient contentment [33-34]. By leveraging virtual microscopy, interactive examination formats, second-opinion consultations, diagnostic ultrasound, 3D models and nuclear medicine imaging, telepathology systems can provide a holistic approach to managing oral and maxillofacial pathologies, ultimately improving patient outcomes and advancing the field of OMP.

## Digital imaging and 3D reconstruction:

## Application of Digital pathology in OMP:

With the growing complexity of medical diagnoses and the increasing workload on pathologists, large datasets can be analyzed by AI, which can identify patterns that human experts may not be able to detect. Techniques such as deep learning, particularly convolutional neural networks (CNNs), are being utilized to automate tasks like cell counting and cancer grading, aiming to enhance diagnostic consistency and patient outcomes. Additionally, advancements in AI are aiding in the early detection and prognosis of oral cancer, with tools like the Mobile Mouth Screening Anywhere (MeMoSA) app being developed to analyze oral cavity images. Despite these advancements, the role of pathologists remains vital for interpreting results and guiding research, highlighting the importance of collaboration between AI technologies and medical professionals. The fields in which digital pathology is applied are listed in Figure 4(see PDF) illustrates four key applications of Digital pathology. Figure 5(see PDF) diagram illustrates the integration of AI and ML in enhancing the accuracy of cancer diagnoses in malignant lesions. Scientific research conferences, diagnosis, education and patient understanding AI and ML can significantly enhance the accuracy of cancer diagnoses in oral pathology through several mechanisms. It highlights key mechanisms, including pattern recognition, data analysis, deep learning applications and automated identification of cancerous regions, demonstrating the overall impact on improving diagnostic precision and patient outcomes in oral pathology.

## AI can assist in diagnosing conditions like Sjogren syndrome and salivary gland tumors in several ways:

[1] Image analysis: Deep learning (DL) models can analyze CT images to detect specific features associated with Sjogren's syndrome, such as fatty degeneration of the salivary gland parenchyma. Studies have shown that the diagnostic performance of these models can be equivalent to experienced radiologists and significantly better than inexperienced ones.

[2] Morphological assessment: ML algorithms can classify malignant salivary gland tumors based on their cytologic appearance. For instance, a recursive partitioning algorithm has been used to classify tumor samples into various morphologic variables, improving the accuracy of pathological diagnoses.

[3] Predicting recurrence: AI models can predict the recurrence of salivary gland malignancies after treatment, which is crucial for patient management and follow-up.

[4] Facial nerve injury prediction: AI can analyze histological, clinical, radiological and cytological data to predict the risk of 7th cranial nerve injury during parotid gland surgery, a significant complication in treating salivary gland tumors.

Overall, AI enhances diagnostic accuracy, aids in risk assessment and supports clinical decision-making in the management of salivary gland diseases.

## Key AI techniques used for diagnosing oral diseases include:

Deep learning: This technique is particularly effective in analyzing complex imaging data and is used for various diagnostic applications in dentistry.

Image analysis: AI algorithms analyze radiographic images to identify and classify oral diseases.

ML: This encompasses various algorithms that learn from data to improve diagnostic accuracy over time.

## The conditions targeted by these AI techniques include:

Dental caries: Detection and classification of carious lesions.

Osteoporosis: Diagnosis through imaging analysis.

Maxillofacial cysts and tumors: Segmentation and classification of cysts and tumors.

Periodontitis and periapical disease: Identification and assessment of periodontal conditions.

Maxillary sinusitis: Detection through imaging techniques [35].

## Challenges and limitations:

The technical challenges in implementing AI for diagnosing oral diseases include:

[1] Quality and quantity data: AI models need large datasets for training and the quality of these datasets can significantly impact model performance. Inconsistent or insufficient data can lead to unreliable outcomes.

[2] Generalizability: AI models trained on specific datasets may not perform well across different populations or settings. Ensuring that models are generalizable to various clinical environments is a significant challenge.

[3] Interpretability: Many AI algorithms, particularly deep learning models, operate as "black boxes," making it difficult for clinicians to understand how decisions are made. This lack of transparency can hinder trust and adoption in clinical practice.

[4] Integration with clinical workflows: Incorporating AI tools into existing clinical workflows can be complex. Ensuring that these tools complement rather than disrupt current practices is essential for successful implementation.

[5] Ethical considerations and regulatory: Addressing ethical concerns related to patient data privacy and navigating the regulatory landscape and AI decision-making are critical challenges that need to be addressed.

[6] Technical expertise: There is often a gap in technical expertise among dental practitioners to effectively utilize and interpret AI tools, which can limit their adoption.

These challenges emphasize the need for on-going research and development to enhance the effectiveness and acceptance of AI in oral disease diagnosis [35]. There are cost and accessibility issues in implementing AI, particularly in healthcare settings. Developing and implementing AI systems can come with a high cost, necessitating substantial investments in technology, data infrastructure and training. Furthermore, data quality and accessibility can present difficulties, as top-notch, labeled datasets are essential for developing successful AI models, but creating such datasets can be a time-consuming and expensive process [36, 37]. Furthermore, disparities in access to advanced technologies may lead to inequities in care, particularly for underrepresented groups [38].

## Current research and future directions:

[1] AI and ML Integration: There is a significant focus on developing AI algorithms for image analysis, which can assist pathologists in diagnosing diseases more accurately and efficiently. This includes the use of deep learning techniques to analyze histopathological images and improve diagnostic precision [36].

[2] Standardization of Protocols: The advancement of the field prompts the need for establishing standardized protocols for implementing AI in pathology. This is essential for ensuring consistency and comparability across studies [39, 40].

[3] Ethical and Regulatory Considerations: Pathology researchers are paying more attention to the ethical considerations involved in utilizing AI, such as data privacy, bias and transparency. Ensuring compliance with regulations like GDPR is also a significant area of emphasis [38, 39, 40- 41].

[4] Integration with Clinical Workflows: There is ongoing research into how Digital pathology and AI can be integrated into existing clinical workflows for enhancing diagnostic processes and patient care [40, 41- 42].

[5] Multi-modal Data Utilization: Researchers are exploring the use of multi-modal data (*e.g.*, combining imaging data with genomic and clinical data) to increase diagnostic accuracy and personalize treatment plans [37, 38-39].

These trends highlight the dynamic nature of Digital pathology and its potential to transform diagnostic practices in healthcare.

## Discussion:

The article thoroughly explores the transformative impact of Digital pathology on the field of OMP. It provides a detailed analysis of the benefits of transitioning from traditional light microscopy to Digital pathology, emphasizing the increased efficiency, quality and safety in pathology labs. The incorporation of Whole Slide Imaging, AI and telepathology has significantly revolutionized OMP, enabling the seamless transfer of high-resolution images, real-time data and remote consultations. The article delves into the specific advancements brought about by the combination of AI and ML in OMP, highlighting how these technologies have automated the analysis of histopathology images, improved pattern identification and enhanced predictive accuracy for cancer diagnosis. It also discusses the potential applications of Digital pathology in various aspects such as conferences, diagnosis, education and patient understanding, providing insights into the effectiveness of Digital pathology in medical education and its potential for 3D imaging technology to create microscopic-level simulations. Furthermore, the article addresses the challenges and limitations associated with the implementation of Digital pathology, including issues related to data management, ethical concerns and the need for regulatory frameworks. It presents a balanced perspective by acknowledging both the significant benefits and the potential obstacles associated with adopting Digital pathology in OMP.

## Conclusion:

In conclusion, advancements in Digital pathology, Whole Slide Imaging, AI and telepathology have revolutionized digital OMP by enhancing diagnostic accuracy and patient outcomes. AI and ML have improved cancer diagnosis and offer promise for early detection, despite on-going challenges in data management, ethics and regulation.

## Figures and Tables

**Table 1:** Studies highlighting key applications of DOMP

**Author**	**Year**	**Study Type**	**Key Findings**	**Applications**
Ho *et al.* (2006) [10]	2006	Pilot Study	Automated WSI is a viable modality for surgical pathology quality assurance (QA).	Image management issues such as pathologist interface, hospital network and lab system integration.
Pantanowitz *et al.* (2011) [11]	2011	Review	WSI enhances specific pathology practices.	Remote frozen section diagnosis, consultation, legal cases, patient care, quality assurance.
Bauer *et al.* (2013) [12]	2013	Validation Study	Diagnostic review by WSI is not inferior to microscope slide review.	Primary diagnosis in surgical pathology.
Araújo *et al.* (2018) [13]	2018	Pilot Study	High performance of WSI for diagnostic purposes in oral pathology.	Main disagreements due to challenging cases or insufficient tissue rather than diagnostic methods.
Niazi *et al.* (2019) [14]	2019	Review	Promising advances in DP via AI.	Technical, ethical and legal questions.
Khalifa *et al.* (2024) [15]	2024	Review	AI is improving diagnostic imaging accuracy, efficiency and personalized healthcare.	Recommendations include investment in AI, ethical guidelines and training for healthcare professionals.
Mc Genity *et al.* (2024) [16]	2024	Review	AI shows high sensitivity and specificity in diagnostic tasks using whole slide images.	More disease areas should be explored in future research.

**Table 2:** Studies highlighting integration of AI and pathology

**Author**	**Year**	**Study type**	**Key Findings**
Robertson *et al.* (2017)[17]	2017	AI in breast cancer diagnosis	AI and deep learning improve diagnostic accuracy and biomarker assessment in breast pathology.
Holzinger *et al.* (2017)[18]	2017	AI and human intelligence integration	Combining AI with human intelligence creates augmented pathologists, addressing challenges in DP.
Koelzer *et al.* (2019)[19]	2019	AI in immune-oncology biomarker assessment	AI and computational approaches enhance the assessment of immune-oncology biomarkers like PD-L1.
Niazi *et al.* (2019)[20]	2019	AI challenges in DP	Emphasized research issues in integrating AI into DP.
Colling *et al.* (2019)[21]	2019	AI challenges in DP	Explored challenges of integrating AI into DP and its impact on pathology.
Gupta *et al.* (2019)[22]	2019	AI methods in DP	Overview of ML and AI applications in DP, highlighting algorithm accuracy and use.
Bizzego *et al.* (2019)[23]	2019	AI validation in DP	DAPPER framework for reproducibility and validation of AI algorithms in DP.
Acs *et al.* (2020)[24]	2020	AI in precision pathology	AI as a key factor for the future of precision pathology, focusing on reliability of AI models.
Cheng *et al.* (2021)[25]	2021	AI development challenges in anatomical pathology	Discussed challenges like data augmentation, algorithm development and regulatory issues in AI.
Ahmed *et al.* (2020)[26]	2020	AI in oral oncology	Review of AI advances in oral oncology and is to pathology, focusing on classification systems.
Mun *et al.* (2021)[27]	2021	Future directions of AI in radiology	Proposed directions for improving CAD performance and radiology services with AI.
